# The Changing Paradigm in Infectious Diseases—Host-Directed Medicine: Implications for the Next Generation of ID Physicians

**DOI:** 10.1093/infdis/jiaf497

**Published:** 2025-09-24

**Authors:** Jatin M Vyas, Simon Feys, Michael K Mansour, Joost Wauters, Frank L van de Veerdonk

**Affiliations:** Vagelos College of Physicians and Surgeons, Columbia University, New York, New York, USA; Medical Intensive Care Unit, Department of Internal Medicine, University Hospitals Leuven, Leuven, Belgium; Department of Microbiology, Immunology, and Transplantation, KU Leuven, Leuven, Belgium; Division of Infectious Diseases, Department of Medicine, Massachusetts General Hospital, Boston, Massachusetts, USA; Department of Medicine, Harvard Medical School, Boston, Massachusetts, USA; Medical Intensive Care Unit, Department of Internal Medicine, University Hospitals Leuven, Leuven, Belgium; Department of Microbiology, Immunology, and Transplantation, KU Leuven, Leuven, Belgium; Department of Internal Medicine and Radboud Center for Infectious Diseases, Radboud University Medical Center, Nijmegen, The Netherlands

**Keywords:** immunotherapy, fungal infections, ID training, immunology

## Abstract

The engagement of medical trainees to the field of infectious diseases has waned over recent years. While the reasons for this decline are multifactorial, one prominent concern is the perception that infectious disease specialists serve merely as gatekeepers of antibiotics. In this forward-looking perspective, we discuss a new paradigm for infectious diseases—host-directed therapies. Supported by the discovery of the mechanism of disease in many infectious diseases, coupled with the ever-expanding armamentarium of immune modulators, we envision that infectious disease physicians will soon be delivering immunotherapy coupled with antimicrobials to achieve cures that were previously impossible.

## Teaser

What is the future of infectious diseases? The authors provide a framework to incorporate our understanding of immunology to evolve how ID clinicians will assess infections and harness the growing arsenal of immune-activators and immunomodulators to improve patient outcomes.

Recent National Resident Matching Program data demonstrate that the number of medical school graduates who seek infectious disease training continues to stagnate, whereas other internal medicine specialties, including cardiology and oncology, have gained in popularity [1]. The Health Resources and Services Administration projects that by 2025, the United States will have fewer than 14 000 infectious disease physicians practicing, falling well short of the more than 15 700 specialists needed to serve our communities adequately [2]. About 80% of the counties in the United States do not have a single infectious disease physician [3]. Europe has about half the number of specialist ID physicians per million inhabitants compared to the United States [4]. As we listen to our current internal medicine residents, we hear three major concerns about entering the infectious diseases field: (1) salary compensation, (2) fatigue from the COVID pandemic, and (3) perception that the principal job of infectious disease physicians is to gatekeep antibiotics. Naturally, these opinions are formed in part by their own experiences as trainees presently but fail to take account of the future of our field. While these challenges are formidable (and addressable), little attention has been paid to the future practice of infectious diseases.

Major advances in life expectancy and good health are primarily attributed to controlling infectious diseases. Recognition of germ theory led to hygienic techniques, which decreased the burden of harmful microbes that afflicted morbidity and mortality of human populations [5]. The widespread use of antibiotics in the 1940s led to a precipitous increase in life expectancy [6]. Similarly, the immunization practice pioneered by Edward Jenner permitted the controlled response of antigens to build immunity prior to introduction to the pathogenic microorganism to modulate disease severity [7]. In fact, some vaccinations led to the complete or partial eradication of deadly diseases, including smallpox and poliomyelitis, respectively. The recognition of the importance of the immune system led to the use of nonpathogen-specific intravenous immunoglobulin to prevent infectious diseases in antibody-deficient patients [8]. Despite the current challenges of emerging novel infectious diseases, antibiotic resistance, and outbreaks of vaccine-preventable diseases—all of which require our urgent attention, what is the next significant advance in infectious diseases? We believe that it will be host-directed strategies.

**Figure 1.**
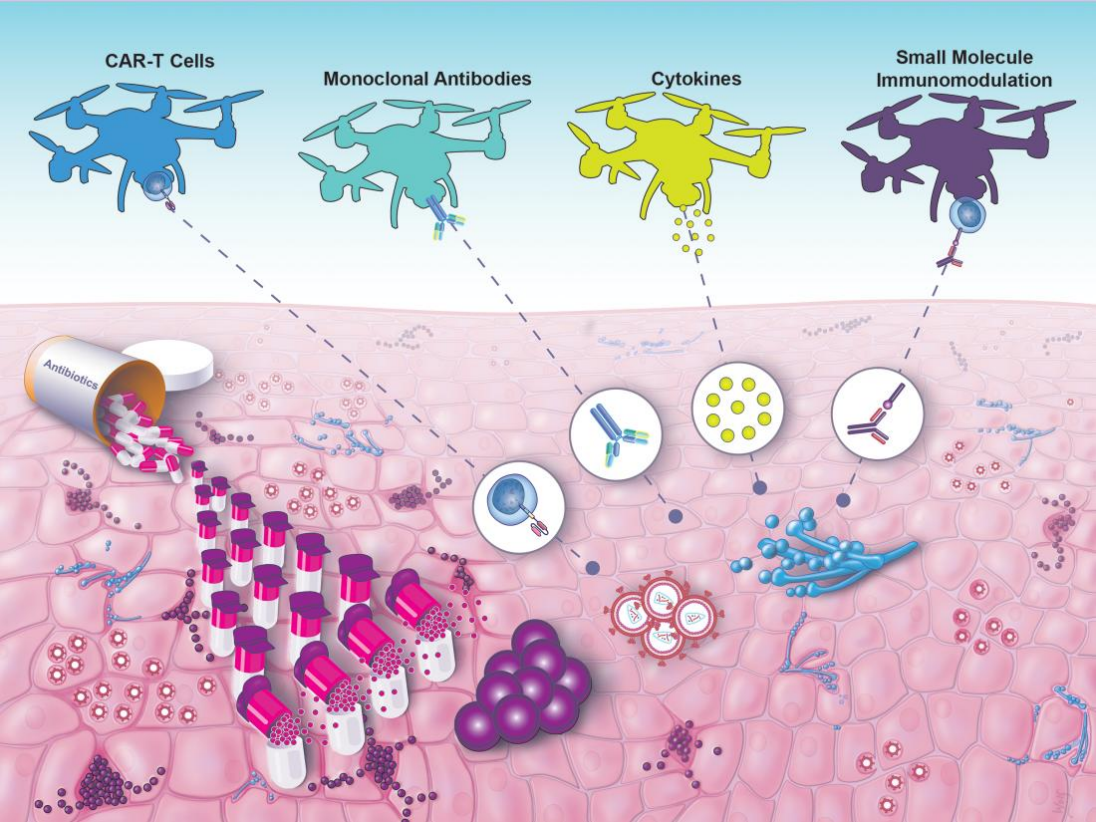
Representation of the battle against infectious diseases. The use of antibiotics (depicted as a ground army) alone is not enough to overcome the battle against invading pathogens. Advances in host-directed weapons (depicted as drones), including CAR-T cells, monoclonal antibodies, cytokine-mediated therapies, and small molecule immunomodulation, are the path forward to support pre-existing forces and defeat the attack of fungi, viruses, and bacteria. Printed with permission from N. Wolf.

The field of infectious diseases has primarily been defined as the clinical practice of microbiology, focusing on microbes. The rules that govern the practice of infectious diseases in immunocompromised patients, including those with hematologic malignancies, recipients of solid-organ transplants, and those taking immune-modulatory medications for cancers or inflammatory disorders, have brought the importance of host–pathogen interactions into sharp focus. Decades of NIH-funded research led to a foundational understanding that the components of the immune system are a critical and necessary partner to achieve optimal outcomes for infections. While patients on immunosuppressive medications represent one type of immunocompromised patient, it became clear that many patients exhibit specific defects of immunity, which render them susceptible to a small number of infections. In parallel, exacerbated immune responses to microbial stimuli also lead to host pathology, oftentimes with deadly consequences, with sepsis and primary ARDS being the most extreme examples. Understanding the genetic and phenotypic characteristics of these patients has led to the concept that further manipulation of the immune system, coupled with conventional antimicrobials, may offer unprecedented opportunities for cures that currently elude many patients (Figure 1).

The theoretical concept that modulating the immune system to effect a cure for human disease does not lie in the unique province of infectious disease. Indeed, immunotherapy is a reality in many fields, including oncology and rheumatology, and has brought dramatic advances. Monoclonal antibody targeting CTLA-4 boosts the CD8+ T cell response, contributing to dramatically improved survival rates in melanoma and lung cancer [9, 10]. Similarly, a complement inhibitor targeting the interaction of the anaphylatoxin C5a with its receptor is highly effective for treating antineutrophil cytoplasmic antibody-associated vasculitis [11]. Recently, NIH investigators detailed the molecular analysis of autoimmune polyendocrine syndrome type 1, a rare autoimmune disorder, which led to the observation that pathogenesis is caused by excessive, multiorgan interferon-γ-mediated responses [12]. These findings led to the application of JAK inhibition in a small cohort of these patients with impressive clinical responses [12, 13]. Has this approach been used in infectious diseases?

## HOST-DIRECTED THERAPIES IN INFECTIOUS DISEASES—A NEW PARADIGM

The three objectives of host-directed therapies include [1] interfering with host cell factors required by a pathogen for replication or persistence [2], enhancing protective immune responses against a pathogen, and [3] reducing pathogen-induced exacerbated inflammation to rebalance immune reactivity at sites of pathology [14]. Embedded in this concept is the notion that immunotherapeutic approaches may be pathogen-specific or nonpathogen-specific; the latter addresses specific defects in immunocompromised patients. Indeed, therapies directed at the host instead of the pathogen are nascent but are now emerging in clinical use. The focus has largely been on difficult-to-treat infections, including viruses, drug-resistant bacterial infections, and invasive fungal infections. A prominent example includes the success of corticosteroids and IL-6 inhibition in the treatment of severe COVID-19 infections [15, 16]; these examples have been extensively discussed elsewhere [17–19]. Cellular therapy in the form of in vitro expanded virus-specific CD8+ T cells for cytomegalovirus and adenovirus infections has been used clinically in immunocompromised patients [20, 21]. Treating a fungal infection with corticosteroids on top of trimethoprim-sulfamethoxazole in severe *Pneumocystis jirovecii* pneumonia, even in non-HIV patients, is standard of care [22]. Several clinical trials encompassing targeting distinct immunological pathways in *Mycobacterium tuberculosis* infection are underway [23]. The role of interferon-γ as a mechanism to enhance immune effector functions in invasive candidiasis has been promising and is currently being trialed [24]. Host-directed therapy can also adopt a personalized approach. Using a novel framework for characterizing the immune response to a refractory infection, Tsai *et al.* designed an immunomodulatory treatment to augment the immune response. They described a case of life-threatening disseminated coccidioidomycosis in a previously healthy child. Immunologic testing showed amplified production of interleukin-4 and reduced production of interferon-γ, indicating an exaggerated type 2 immune response. Supplementation of antifungal agents with interferon-γ treatment slowed disease progression, and the addition of interleukin-4 and interleukin-13 blockade with dupilumab resulted in rapid resolution of the patient's clinical symptoms [25].

The frameshift in perspective is to dethrone the primacy of the invading microorganism and recognize that the following equation better captures the problem to solve in clinical medicine:

Microorganism + Immune response = Clinical presentation/status.

Currently, we place great emphasis on determining the identity of the pathogen and its characteristics. As we choose a course of antimicrobial therapy, we must consider the immune status of the patient (presumed immunocompetent vs. immunocompromised). Still, little is performed to determine the nature and quantitative response of the immune response, as well as the efficacy of this response. The theoretical basis for the equation above is extensively defined throughout the damage-response framework [26]. However, more steps must be taken to implement this fully in clinical practice.

## TOOLS NEEDED FOR IMPLEMENTATION

**Figure 2.**
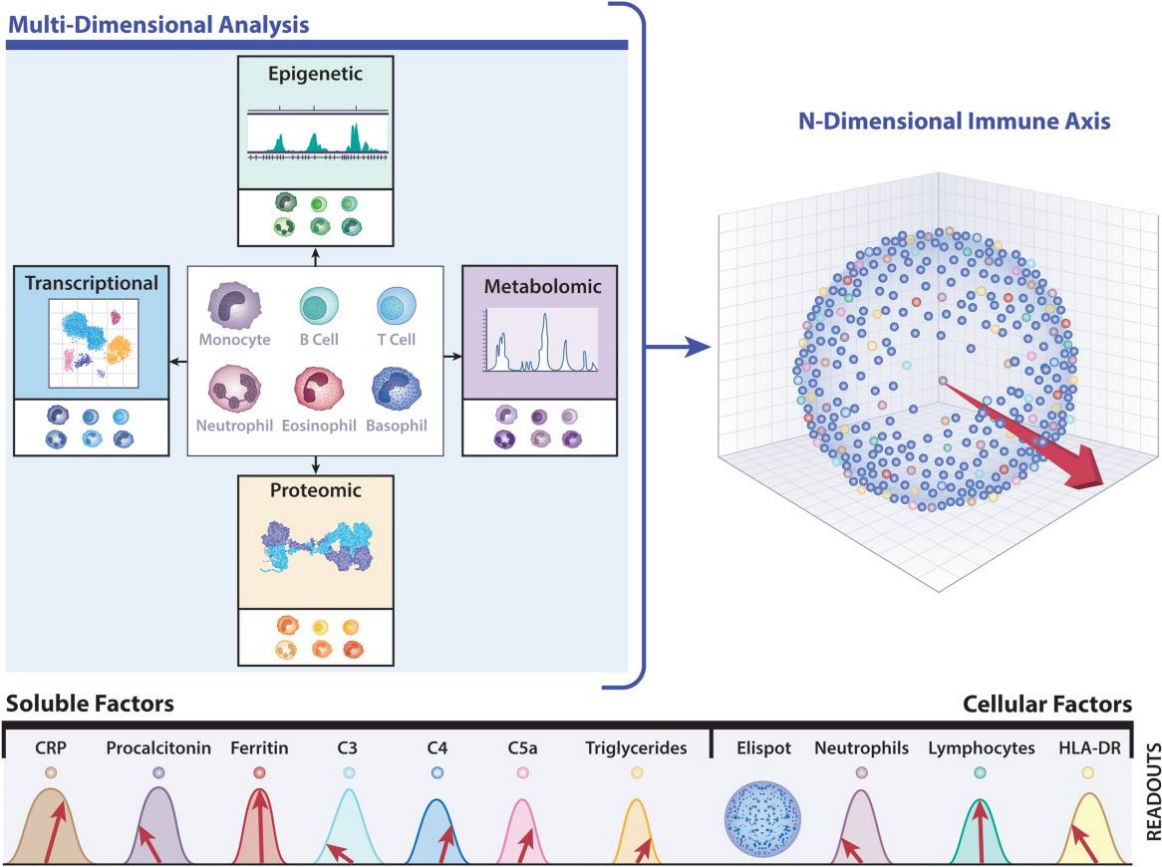
The immune-vector conceptualizes the immune response to infection in individual patients and is derived from the interrogation of multiple measurable immune parameters. During infection, the immune system changes over time, and this can be conceptualized as a dynamic “immune vector”—similar to the integration of multiple electrical vectors that comprise an electrocardiogram. While a small fraction of immune biomarkers are readily available for use by clinicians, ongoing research seeks to define additional parameters using, but not limited to, transcriptomics, proteomics, metabolomics, and epigenetics. While achievable in research laboratories, these readouts are not yet available for routine use in hospitals. By assessing the preintervention state of the immune system, we could better tailor treatments and monitor how the immune system responds to the invading pathogen(s). This approach would lead to a more personalized and effective treatment for infectious diseases. Printed with permission from N. Wolf.

Currently available clinical tests do not interrogate the immune system with sufficient resolution to predict either risk for severe complications of infection or sterilization and resolution of infection. However, preclinical host–pathogen interaction research has provided quantitative and functional characterization of the immune response, and we anticipate that these will be converted into clinical practice in the future to create a dynamic immune-vector specific for each patient (Figure 2). In analogy with the real-time heart vector underlying an ECG, the immune vector travels around in the n-dimensional immune landscape, shaped and reshaped by data delivered by clinically available biomarkers of inflammation, use of single-cell detection technique that quantifies the frequency of immune cells secreting specific proteins (Elispot) [27], coupled with multiparametric flow cytometry, including intracellular cytokine staining, single-cell transcriptomics, proteomics, metabolite profiling, and epigenetic immune cell quantification. Adapted for clinical purposes, this immune vector concept will provide unprecedented insights into the pathogen-induced immune response in real time. Individually, high-throughput omics approaches provide a window into immune responses and phenotypes that portend increased risk of infection and may foretell clinical course [28]. Given the complex interplay among different aspects of the immune system, combining multiomics modalities into a computational framework increases predictive power and reveals crosstalk between various layers of biological profiling, approaching the development of digital twins to inform a rational approach to immune modulation combined with antimicrobials. Taking this one step further, we envisage designing functional tests using immune cells that capture this full complexity, providing a platform for personalized *ex vivo* immune modulation testing for individual patients at specific time points.

## OUR VISION FOR THE FUTURE

There is hubris in believing humans will win the antimicrobial arms race against pathogens. Host–pathogen studies provide a novel approach to the treatment of infectious diseases. Host-directed therapies promise unprecedented treatments for difficult-to-treat infections without the potential collateral damage of promoting antimicrobial resistance and indiscriminate damage to the host and host microbiome. The infectious disease specialist of the future will need to understand the host biology of infection and develop the ability to interpret immune status, skills that should be incorporated into updated undergraduate and graduate medical education programs. Moreover, grounded by the discovery of the mechanism of disease in many infectious diseases, coupled with the ever-expanding armamentarium of immune modulators, we envision that infectious disease physicians will soon be delivering immunotherapy coupled with antimicrobials to effect cures when none have been possible. Small molecules and immune effectors will allow physicians to dial down or dial up the immune response. We will have new tools to augment responses to eliminate pathogens for patients whose immune response is insufficient. We can modulate the immune response to restore homeostasis for patients whose pathology is derived from pathogen-induced hyperinflammation.

## SUMMARY

Despite real challenges in the field of infectious diseases that dampen the enthusiasm of trainees to join this workforce, we see an exciting future for our field thanks to foundational research and the ability to translate these findings into new tools to guide infectious disease physicians to both modulate the immune response and target pathogens with antimicrobials to improve patient outcomes.
